# Association between serum lipoprotein levels and neurological function in patients with acute ischemic stroke: A protocol of systematic review and meta-analysis

**DOI:** 10.1097/MD.0000000000020258

**Published:** 2020-05-15

**Authors:** Yao-Jia Jiang, Zeng-Mian Wang, Ze-Yu Wang, Chun-Jie Wei

**Affiliations:** Third Ward of Neurology Department, First Affiliated Hospital of Jiamusi University, Jiamusi, China.

**Keywords:** acute ischemic stroke, association, neurological function, serum lipoprotein levels

## Abstract

**Background:**

The target of this study is to summarize the association between the serum lipoprotein levels and neurological function in patients with acute ischemic stroke.

**Methods:**

A comprehensive search of Cochrane Library, PUBMED, EMBASE, Web of Science, and Chinese Biomedical Literature Database, China National Knowledge Infrastructure from inception to the February 29, 2020 without language and publication date restrictions. All searched studies will be selected by 2 authors independently against the eligibility criteria. Included studies will be critically appraised, and essential data will be extracted by 2 independent authors. If necessary, meta-analysis will be utilized to synthesize the outcome data from included articles. If it is not possible, a narrative synthesis will be undertaken.

**Results:**

This study will summarize the up-to-date evidence to investigate the association between serum lipoprotein levels and neurological function in patients with acute ischemic stroke.

**Conclusion:**

Its results may present beneficial evidence and guidance for the clinical practice and further studies.

**Study registration number:**

INPLASY202040043.

## 1 Introduction

Acute ischemic stroke (AIS) is the most type of stroke,^[1,2,3,4,5]^ which accounts for about 87% of all stroke population.^[6]^ It occurs when blood flow is blocked by a clot, which limits the blood supply to the brain.^[7,8,9]^ Several risk factors contribute to such disorder, including hyperextension, diabetes, heart diseases, smoking, age and gender, family history of stroke, and brain aneurysms or arteriovenous malformations.^[10,11,12,13,14,15]^ If such disorder cannot be treated fairly well, it may cause a variety of sequelae in stroke survivors, which significantly affect their quality of life.^[16]^

Previous study reported that serum lipoprotein has a certain predictive effect on the long-term clinical prognosis in AIS survivors,^[17]^ which plays a very essential role in AIS recovery, especially in AIS survivors with neurological function (NF).^[18]^ Several studies reported that serum lipoprotein levels (SLL) is closely associated with NF in AIS.^[18,19,20,21]^ However, no study has been conducted at the evidence-based medicine level. Thus, this study will firstly explore the association between SLL and NF in AIS survivors.

## 2 Methods

### 2.1 Study registration

This protocol has been funded and registered on INPLASY202040043, and has been organized according to the Preferred Reporting Items for Systematic Reviews and Meta-Analysis (PRISMA) Protocol statement guidelines.^[22]^

### 2.2 Study selection criteria

#### 2.2.1 Study types

All case-controlled studies on investigating the associations between SLL and NF will be included with no limitations of language and publication time.

### 2.3 Types of exposures

All participants in the experimental group had AIS.

All subjects in the control group were health participants without AIS.

### 2.4 Population

We will include participants who were diagnosed as AIS, or normal healthy subjects, irrespective their country, race, gender, and age.

### 2.5 Outcomes

The primary outcomes are serum lipoprotein levels, as detected by enzyme linked immunosorbent assay, and NF, as measured by National Institutes of Health Stroke Scale or other relevant scales.

The secondary outcomes are fasting blood glucose, triglycerides, total cholesterol, low density lipoprotein cholesterol, high density lipoprotein cholesterol, systolic blood pressure, diastolic blood pressure, and uric acid.

### 2.6 Search strategy

We will comprehensively search the following electronic databases of PUBMED, EMBASE, Cochrane Library, Web of Science, and Chinese Biomedical Literature Database, China National Knowledge Infrastructure from inception to the February 29, 2020 with no language and publication date restrictions. The detailed search strategy for PUBMED is summarized (Table 1). Search strategies for other electronic databases will be modified based on this strategy.

**Table 1:**
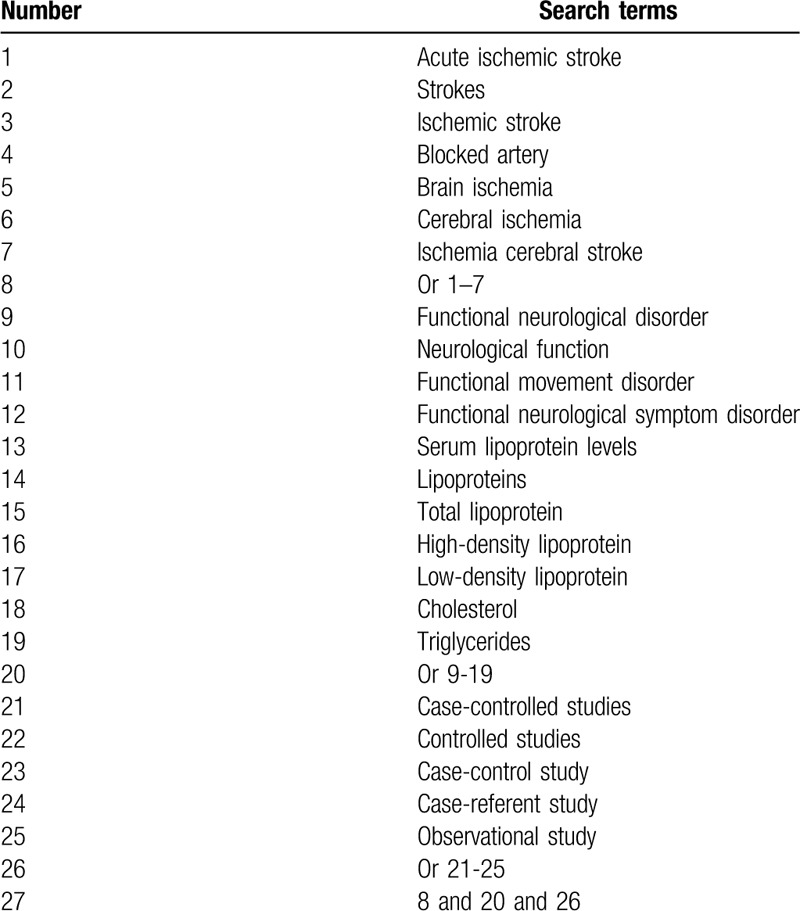
Search strategy for PubMed.

In addition, relevant conference abstracts, ongoing trials from clinical trial registries, and reference lists of included studies will be searched.

### 2.7 Study selection

After duplicates removed, 2 authors will independently examine the tiles/abstracts of searched literatures to eliminate any unrelated studies. Then, we will obtain full papers of potential studies to determine whether they fulfill all inclusion criteria. Disagreements will be solved by consensus with the help of another author. The process of study selection will be shown in a PRISMA flow chart.

### 2.8 Data extraction and management

Two independent authors will perform data extraction from all eligible studies. Any confusion will be cleared up through discussion with the help of another author. For each included study, the following data will be collected: title, first author, time of publication, country, participant's age, gender, race, severity of AIS, study setting, study design, sample size, outcomes, follow-up information, results findings, and conflict of interest.

### 2.9 Study quality assessment

Study quality of each included article will be assessed by 2 independent authors using Newcastle-Ottawa Scale.^[23]^ Any doubt between 2 authors will be figured out by consulting another experienced author, and a consensus will be reached.

### 2.10 Unit of analysis

If this study includes cross-over articles, only the data from the first study period will be used.

### 2.11 Missing data dealing with

Any unclear or missing data will be requested from primary authors through email. We will analyze the available data using intention-to-treat analysis if we cannot obtain the insufficient or missing data. We will discuss its impacts as a study limitation.

### 2.12 Data synthesis

We will employ RevMan 5.3 software to synthesize and analyze data. All continuous data will be recorded as mean difference or standardized mean difference and 95% confidence intervals, while all dichotomous data will be calculated as risk ratio or rate ratio and 95% confidence intervals. We will examine the statistical heterogeneity using *I*^2^ test. *I*^2^ ≤ 50% suggests homogeneity, and a fixed-effects model will be utilized. *I*^2^ > 50% shows remarkable heterogeneity, and a random-effects model will be employed. If homogeneity across sufficient studies is identified, we will perform a meta-analysis. If remarkable heterogeneity among eligible studies is examined, we will undertake a subgroup analysis to explore the sources of obvious heterogeneity. Whenever necessary, we will also conduct a narrative summary.

### 2.13 Subgroup analysis

If data are available, a subgroup analysis will be performed to detect sources of obvious heterogeneity regarding the types of participants, and outcome indicators.

### 2.14 Sensitivity analysis

In the case of sufficient data, a sensitivity analysis will be conducted to test the robustness of study findings regarding the methodological quality and missing data.

### 2.15 Publication bias

We will carry out a funnel plot, Egger regression and Begger tests to check if there are any publication biases when at least 10 studies are included.^[24,25]^

### 2.16 Ethics and dissemination

This study does not need research ethics approval because of no confidential patient data will be used. Its results will be disseminated on a peer-reviewed journal or via a conference presentation.

## 3 Discussion

A variety of published studies reported the association between SLL and NF in patients with AIS. However, no systematic review has specifically conducted the association between SLL and NF in patients with AIS. This study will firstly summarize rigorous evidence of the association between SLL and NF in patients with AIS among all included studies. The results of this study will inform the understanding of the association between SLL and NF in patients with AIS.

## Author contributions

**Conceptualization:** Yao-Jia Jiang, Ze-Yu Wang, Chun-Jie Wei.

**Data curation:** Yao-Jia Jiang, Zeng-Mian Wang, Ze-Yu Wang, Chun-Jie Wei.

**Formal analysis:** Yao-Jia Jiang, Ze-Yu Wang.

**Funding acquisition:** Chun-Jie Wei.

**Investigation:** Chun-Jie Wei.

**Methodology:** Yao-Jia Jiang, Zeng-Mian Wang, Ze-Yu Wang.

**Project administration:** Chun-Jie Wei.

**Resources:** Yao-Jia Jiang, Zeng-Mian Wang, Ze-Yu Wang.

**Software:** Yao-Jia Jiang, Zeng-Mian Wang, Ze-Yu Wang.

**Supervision:** Chun-Jie Wei.

**Validation:** Yao-Jia Jiang, Zeng-Mian Wang, Ze-Yu Wang, Chun-Jie Wei.

**Visualization:** Yao-Jia Jiang, Ze-Yu Wang, Chun-Jie Wei.

**Writing – original draft:** Yao-Jia Jiang, Zeng-Mian Wang, Ze-Yu Wang, Chun-Jie Wei.

**Writing – review & editing:** Yao-Jia Jiang, Zeng-Mian Wang, Chun-Jie Wei.
